# Brachytherapy versus radical hysterectomy after external beam chemoradiation: a non-randomized matched comparison in IB2-IIB cervical cancer patients

**DOI:** 10.1186/1477-7819-7-19

**Published:** 2009-02-16

**Authors:** Lucely Cetina, Alicia Garcia-Arias, Myrna Candelaria, David Cantú, Lesbia Rivera, Jaime Coronel, Blanca Bazan-Perkins, Vladimir Flores, Aaron Gonzalez, Alfonso Dueñas-González

**Affiliations:** 1 Division of Clinical Research, Instituto Nacional de Cancerología (INCan), Mexico City, Mexico; 2 Department of Gynecological Oncology, INCan, Mexico City, Mexico; 3 Division of Radiation Oncology, INCan, Mexico City, Mexico; 4 Unit of Biomedical Research on Cancer, Instituto de Investigaciones Biomédicas (IIB), Universidad Nacional Autónoma de México (UNAM)/INCan, Mexico City, México

## Abstract

**Background:**

A current paradigm in the treatment of cervical cancer with radiation therapy is that intracavitary brachytherapy is an essential component of radical treatment. This is a matched retrospective comparison of the results of treatment in patients treated with external beam chemoradiation (EBRT-CT) and radical hysterectomy versus those treated with identical chemoradiation followed by brachytherapy.

**Methods:**

In this non-randomized comparison EBRT-CT protocol was the same in both groups of 40 patients. In the standard treated patients, EBRT-CT was followed by one or two intracavitary Cesium (low-dose rate) applications within 2 weeks of finishing external radiation to reach a point A dose of at least 85 Gy. In the surgically treated patients, radical hysterectomy with bilateral pelvic lymph node dissection and para-aortic lymph node sampling were performed within 7 weeks after EBRT-CT. Response, toxicity and survival were evaluated.

**Results:**

A total of 80 patients were analyzed. The patients receiving EBRT-CT and surgery were matched with the standard treated cases. There were no differences in the clinicopathological characteristics between groups or in the delivery of EBRT-CT. The pattern of acute and late toxicity differed. Standard treated patients had more chronic proctitis while the surgically treated had acute complications of surgery and hydronephrosis. At a maximum follow-up of 60 months, median follow-up 26 (2–31) and 22 (3–27) months for the surgery and standard therapy respectively, eight patients per group have recurred and died. The progression free and overall survival are the same in both groups.

**Conclusion:**

The results of this study suggest that radical hysterectomy can be used after EBRT-CT without compromising survival in FIGO stage IB2-IIB cervical cancer patients in settings were brachytherapy is not available. A randomized study is needed to uncover the value of surgery after EBRT-CT.

## Background

Cervical cancer ranks seventh in the list of most frequent cancers worldwide. However, this tumor is second only after breast cancer as the most common gynecological neoplasm [1]. Currently, chemoradiation is accepted as the standard of care for patients with locally advanced cervical cancer. An updated meta-analysis that includes 4,921 patients shows that chemoradiation improves overall survival and progression-free survival, whether or not platinum is used, with absolute benefits of 10 and 13%, respectively [2].

There exists some lack of consensus on what early stage and locally advanced stages are. Thus, in Institutions that routinely employs radical hysterectomy to treat IB1, IB2 and IIA stages the definition of locally advanced stages encompasses from IIB to IVA as these later are treated with chemoradiation as definitive therapy. On the contrary, Institutions that use chemoradiation for IB2 and IIA cases defines locally advanced disease as stages IB2-IVA. In this context it is now clear that regardless of the Institutions treatment preference, the treatment of early stage patients can be done by radical hysterectomy or definitive radiation. This evidence is supported by an early [3] and a more recent randomized trial [4], in which 343 eligible patients were randomised to either surgery with or without adjuvant radiation or definitive radiation. After a median follow-up of 87 (range 57–120) months, 5-year overall and disease-free survival were identical in the surgery and radiotherapy groups.

A current paradigm in the treatment of cervical cancer with radiation therapy is that intracavitary brachytherapy is an essential component of radical treatment of cervical cancer. On the other hand, despite that for many years many institutions routinely used adjuvant extrafacial hysterectomy for bulky exophytic or "barrel" shaped tumors, this procedure has been gradually abandoned as a randomized study shows no benefit on survival of adjuvant hysterectomy, despite the study suggests that patients with tumors measuring 4 to 6 cm, may benefit from extrafascial hysterectomy [5].

A well-known prognostic factor in cervical cancer is the pathological complete response achieved with preoperative treatment [6]. It is at least intriguing that a retrospective comparison of the complete pathological response rates in patients undergoing adjuvant surgery after primary external beam radiation with and without brachytherapy shows no differences [7]. Overall, in trials using external beam radiation (EBRT) at doses between 37.4 to 52 Gy, in common fractions of 1.8 or 2 Gy daily, plus brachytherapy the average complete pathological complete response rate observed is 50% (41%, 44%, 48%, 48%, 69%) [8-11], whereas in those using EBRT-CT at similar doses with either weekly cisplatin or the combination of cisplatin and 5-fluorouracil plus brachytherapy, the corresponding average is 51.1% (38%, 45%, 49%, 52%, 60% and 63%)[11-16]. Interestingly, in four trials (one of them with to arms) using EBRT-CT but no brachytherapy, the pathological response rate is essentially the same, a mean of 51.6% (45%, 45.2%, 46.6%, 54.2 and 67%) [17-20]. These data are quite provocative and suggest that for these stages, brachytherapy could be dispensable, however, it must be stressed that such comparison is based on highly heterogeneous trials and as such data is only hypothesis generating. To gain further insight into this issue we have performed a retrospective comparison of the results of treatment in patients treated with chemoradiation and radical hysterectomy versus those treated with standard chemoradiation (external beam and brachytherapy).

## Patients and methods

This is a non-randomized retrospective comparison of two groups of patients treated with standard chemoradiation (external beam and brachytherapy) or preoperative chemoradiation (external beam and radical hysterectomy). The surgically treated patients were those in the cisplatin arm reported in the study *"Pathologic response and toxicity assessment of chemoradiotherapy with cisplatin versus cisplatin plus gemcitabine in cervical cancer: a randomized Phase II study" *which was performed between May 1999 and June 2000, and included 83 patients, 40 to cisplatin and 43 to cisplatin gemcitabine during external radiation [21].

These 40 patients were matched with 40 patients out of 294 who received radiotherapy and concurrent cisplatin at our Institution between January 1999 and December 2003 as reported [22]. Firstly they were matched by age, then for FIGO stage and then for histology once these three criteria were met the selected 40 patients were compared. Patients in both cohorts had a histological diagnosis of cervical carcinoma and were staged according to the FIGO classification using standard pre-treatment workup [21].

### Treatment

Patients received external beam radiation using megavoltage machines (Co^60 ^or lineal accelerator equipment) with a minimum photon-beam energy of 2.25 MV with an isocenter technique to the whole pelvis for a total dose of 50 Gy (5 weeks, 2 Gy fractions from Monday to Friday) followed by one or two intracavitary Cesium (low-dose rate) applications within 2 weeks of finishing external radiation. The planned total dose to point A was 85 Gy. Patients were treated with the conventional 4-field box technique. Irradiated volume was to include the whole uterus, paracervical, parametrial, and uterosacral regions, as well as external iliac, hypogastric, and obturator lymph nodes.

Cisplatin was administered for 6 weeks during external radiation, beginning on the first day of radiation. Cisplatin infusion was administered within 2 h either before or after radiation application. A dose of 40 mg/m^2 ^(maximum dose, 80 mg) was used and administered via a peripheral vein to patients in an out-patient setting as follows: 1,000 mL of normal saline for 1 h followed by cisplatin diluted in 500 mL of normal saline containing 62.5 mL of 20% mannitol for 1 h, followed by 500 mL of normal saline for 30 min. Intravenously (i.v.), 8 mg of dexametasone and 8 mg of ondansetron were employed as antiemetic prophylaxis. Cisplatin (but no radiation) was withheld in any case involving grade 3 toxicity until the toxicity regressed to any grade of <3; in patients with grade 3 toxicity that persisted >2 weeks, chemotherapy was no longer administered. Radiation was only stopped in cases of grade 4 hematologic or non-hematologic toxicity until toxicity resolved to at least grade 3.

In the patients treated with standard protocol, external radiation was followed by one or two intracavitary Cesium (low-dose rate) applications within 2 weeks of finishing external radiation to reach a point A dose of at least 85 Gy.

In the surgically treated patients, type III radical hysterectomy with bilateral pelvic lymph node dissection and para-aortic lymph node sampling were performed within 7 weeks after external chemoradiation. In addition, postoperative brachytherapy was performed in cases with one or more high-risk factors for recurrence: positive surgical margins, positive pelvic lymph node and residual disease in parametria as well as those cases with any intermediate-risk factor for recurrence: vascular or lymphatic permeation and deep of invasion to the middle or internal thirds of the cervical stroma. An exception to this was the cases with isolated positive pelvic nodes without any other high or intermediate-risk factor. Brachytherapy was administered within 4 weeks after surgery using Cesium sources at a dose of 30–35 Gy to the vaginal mucosa delivered to a deep of 0.5 cm.

### Evaluation of toxicity

The acute and chronic toxicities of treatments were evaluated according to the RTOG toxicity criteria.

### Survival

Patients were followed with every three months visits in which a complete and pelvic examination as well as blood counts, clinical chemistry and chest X-rays were performed. CT scans, ultrasounds and other studies were done when appropriate. Survival and progression-free survival were calculated in an intention-to-treat and they were considered from the date of diagnosis until death or the last visit. Curves were constructed using the Kaplan-Meier method [23] and the log-rank test[24] to assess differences between groups. The chi-square and *t *tests were used when appropriate to compare patient characteristics, responses and toxicity. The randomized phase II study from which this data is taken retrospectively was approved by IRB.

## Results

### Characteristics of patients

A total of 80 patients were analyzed retrospectively. The patients receiving EBRT-CT and surgery were matched with the standardly treated cases. The clinical characteristics of the two groups of patients are shown in Table 1. Clinicopathological characteristics were well-balanced and there were no differences in age, histology, stage, tumor size, parametrial infiltration, hemoglobin levels and performance status. In addition (not shown), the socioeconomic and demographic status were also similar in both group of patients (Table 1).

**Table 1:** Baseline characteristics.

Clinicopathological		surgery	brachytherapy
Number		40	40

Karnofsky status		90	90

Age (median)		45 (24–70)	45 (24–70)

Stage			

	IB2	9 (22%)	9 (22%)

	IIA	4 (10%)	4 (10%)

	IIB	27 (68%)	27 (68%)

Histology			

	Squamous	28 (70%)	

	28 (70%)		

	Adenocarcinoma	8 (20%)	8 (20%)

	Adenosquamous	4 (10%)	4 (10%)

Hemoglobin gr/dL			

	Median	14.4 (10.1–15.3)	13.4 (10.4–15.2)

	Tumor size (cm2)	32.5 (16–81)	34 (16–84)

Table 2 shows the details of external chemoradiation in both arms. The median number of cisplatin courses administered was 6 (5–6). The mean dose of external radiation was 50 Gy in both arms (38–56 and 46–56) and the time to complete radiation was 39 (34–59) and 41 (25–81) days respectively. In the group treated with brachytherapy, the mean dose to point A was 82.57 (74–88 Gy).

**Table 2:** Treatment in both groups.

		Surgery	Brachytherapy
Number		40 (12 received ADJ. Brachy.)	40

Completed treatment		40	40

Median # of cycles		6 (5–6)	6 (5–6)

	6 cycles	82%	80%

	5 cycles	18%	20%

Median dose EBRT (Gy)		50 (34–59)	50 (46–56)

Time to complete EBRT (d)		39 (34–59)	41 (25–61)

Dose to point A (Gy)			82.6 (74–88)

External chemoradiation by group. ADJ: adjuvante brachytherapy was performed in 30% of patients in the surgical arm.

### Toxicity

Acute toxicity to external beam chemoradiation was similar in both groups (not shown) as treatments were the same and already reported [21,22]. While in the group of patients receiving standard brachytherapy no acute complications were observed, in the surgical arm, the following acute complications were observed: postoperative infections were seen in three patients (2 urinary, 1 pulmonary). One patient had wound dehiscence and another developed an intraabdominal abscess. In the late (after 30 days) postoperative period, five patients coursed with mildly symptomatic (low-grade fever and pain) unilateral lymphocysts that required treatment. Three cases resolved with percutaneous drainage, however, two patient required lymphocyst resection and drainage. In addition, one patient had an ureterocutaneous fistula and four patients developed uni or bilateral hydronephrosis which required some form of urinary drainage [21]. Regarding late toxicity, table 3 shows, however, that profile of late toxicity was different. In the surgery group there were 6 patients with hydronephrosis (three grade 1 – defined as *unilateral ureteral obstruction, not requiring surgery*- and three grade 2 – defined as *bilateral ureteral obstruction, not requiring surgery*-) whereas no events were registered in the standardly treated arm, (p < 0.016). None of these patients had clinical data of renal function deterioration, the levels of creatinine remained within the normal range. On the contrary, proctits was more common in the group of patients receiving standard EBRT-CT and brachytherapy. There were one and three patients suffering from grades 1 and 2 proctitis respectively in the surgery arm whereas 10 patients from the EBRT-CT brachytherapy group had grade 2, and one patient each having grade 1, grade 2 and grade 4 proctitis, in total they were 4 versus 13 patients with the event respectively (p < 0.008). There were no differences however in the frecuency and severity of cystitis (p = 0.785).

**Table 3:** Late toxicity.

Status	surgery				brachytherapy				
**Toxicity/Grade**	**1**	**2**	**3**	**4**	**1**	**2**	**3**	**4**	

Hydronephrosis	3	3	0	0	0	0	0	0	p < 0.016

Proctitis	1	3	0	0	1	10	1	1	p < 0.008

Cystitis	0	1	2	0	0	0	2	1	p = 0.785

Hydronephrosis was graded according to the CTC NCI version 2 whereas proctitis and cystitis according to the RTOG/EORTC Late Radiation Morbidity Scoring Scheme (Appendix IV of the CTC NCI version 2).

### Response and survival

In the surgical arm, 22 (55%) patients had a pathological complete response. Among the 18 partial responders, 7 patients had either positive pelvic nodes or positive surgical margins (high-risk factors), seven had a combination of high and intermediate-risk factors and four patients had only either a deep stromal invasion and/or lymphovascular permeation. All of them were intended to receive postoperative brachytherapy, however, only 12 out of the 40 patients (30%) actually were treated with postoperative brachytherapy. The median dose of brachytherapy for all patients receiving this treatment was 33.24 Gy (28.6–35.9). In the standard treatment group all patients completed both, external and intracavitary therapy and the complete response rate was 85% (34 out of 40).

**Figure 1:**
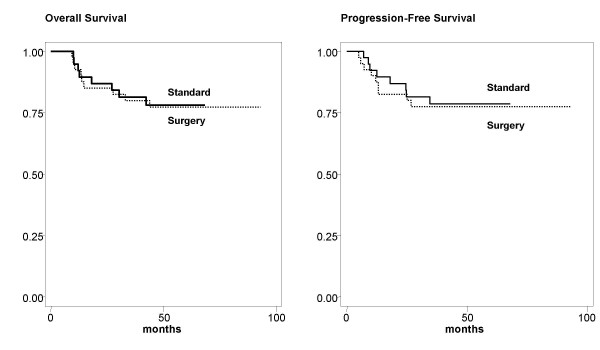
**Disease-Free and Overall survival rates for both groups are the same**.

At a maximum follow-up of 60 months, median follow-up 26 (2–31) and 22 (3–27) months for the surgery and standard therapy groups the progression free and overall survival are similar with a projected 5-year survival of 78%. Eight patients per group have recurred and died of disease, Figure 1. In regard to the pattern of relapses, Table 4 shows that 8 patients relapsed in the both groups respectively. Of note, in the brachytherapy, there were 5 pelvic alone, one pelvic/systemic and one pelvic/retroperitoneal whereas in the surgical arm there were only 4 who have pelvic relapse and one pelvic/systemic.

**Table 4:** Patterns of relapse in both groups.

	Surgery	Brachytherapy
Pelvic alone	4 (6,8,10,12 m)	5 (4,7,7,12,23 m)

Systemic alone	2 (12, 30 m)	1 (12 m)

Pelvic and RTP	1 (12 m)	1 (8 m)

Pelvic and systemic	none	1 (6 m)

RTP alone	1 (4 m)	none

Sites and time of relapse. RTP: retroperitoneal; m: months.

## Discussion

Available evidence in literature indicates that early stage bulky tumors can be managed with either primary chemoradiation (external and brachytherapy) or with radical hysterectomy and tailored adjuvant radiation or chemoradiation. Both approaches have pros and cons which have been widely commented in literature [25-28]. On the contrary, there are few reports that have evaluated the role of radical hysterectomy after either external beam radiation or chemoradiation at doses ranging from 37.4 to 52 Gy with or without brachytherapy.

The value of radical hysterectomy after primary radiation or chemoradiation has not been evaluated in randomized trials. A study was reported in 1993 in which 20 uterine cancer patients with bulky disease FIGO staged as I (50%), II 45% and 5% stage III, deemed to have at high risk for recurrence underwent radical hysterectomy after definitive radiation. Authors conclude that radical hysterectomy after radiation is morbid but may be effective in treating patients with 1) large cervical tumors, 2) cervical cancer that responds poorly to radiation, 3) small recurrent cervical tumors, 4) patients unable to undergo brachytherapy for cervical cancer, and 5) uterine sarcomas involving the cervix[29]. In a larger study of 187 patients, radiotherapy-followed by radical surgery including systematic para-aortic lymphadenectomy was evaluated. While there were complications in 18% of patients, overall survival at 3 years was 85%, 56%, and 40% in patients with negative nodes, positive pelvic nodes, and positive para-aortic nodes, respectively [30]. In a more recent study in 30 patients it was found that adjuvant surgery may improve the outcome of patients with bulky residual tumor after chemoradiation for locally advanced cervical cancer, allowing a 5-year survival of 55.6% after curative intervention [31]. Another study however, reported that only two out of 10 patients, remained disease-free at a median follow-up of 22 months [32].

The results of this non-randomized comparison suggest that external beam chemoradiation using cisplatin followed by brachytherapy or a radical hysterectomy and tailored brachytherapy offers the same survival probability. Even though the methodological limitations of this type of studies are obvious, a rigorous matching of patients to minimize known biases was done. These results along with the existing data in literature are very suggestive that brachytherapy may be dispensable in early stage cervical cancer patients as long a radical hysterectomy with pelvic lymphadenectomy is performed. An additional potential advantage of surgical treatment is the evidence that after definitive radiation between 11 and 20% of patients are left with positive pelvic lymph nodes that remain untreated if not are removed by surgery [15,33,34]. Yet this triple modality seems promissory regarding local control and survival, surgical complications, specifically lymphocysts, fistula and hydronephrosis are more frequent to that reported in patients undergoing either upfront hysterectomy[35,36] or after neoadjuvant chemotherapy[37]. This higher surgical complication rate of this modality, therefore, should be weighed against the higher frequency of chronic proctitis that is observed when patients receive brachytherapy as standard treatment. It is currently known that cervical cancer survivors treated with radiotherapy had worse sexual functioning than did those treated with radical hysterectomy and lymph node dissection and that operated patients can expect overall quality of life and sexual function not unlike that of peers without a history of cancer[38]. Nevertheless, in that study patients were excluded if they received a combination of surgical and radiation therapies or had concurrent chemoradiotherapy therapy. Hence, this is an important issue which is being addressed in our prospective study as in the modality of therapy here discussed, most patients in the surgical arm receive only a "moderate" dose of external radiation (50 Gy) whereas in the brachytherapy arm, patients receive full radiation dose, (external beam and brachytherapy).

The implications of this study seems obvious. As for now, external beam radiation and brachytherapy remain as the core treatment for most stages of cervical carcinoma. There are some indications that an external beam boost is a reasonable but not optimal option after external beam radiotherapy to the pelvis when it is not possible to perform brachytherapy [39], however, it has been reported that actuarial risk of major complications is greater for patients receiving >52 Gy of EBRT to the central pelvis (57–68%), compared with those who had 48–52 Gy (28%) [40]. In addition, it has been suggested that for achieving an adequate external boost it is required to use high-tech IMRT or IMPT (proton therapy [41] which is not available in many centers.

In many developing countries cervical cancer patients may receive a suboptimal therapy because of poor brachytherapy resources [42-44]. In this situation, external beam radiation followed by a radical hysterectomy could reduce the need of brachytherapy, although the role of an external radiation boost should also be studied, particularly using IMRT or IMPT

## Conclusion

Access not only to radiation but to cancer treatments is one of the areas of greatest need in the developing word. If preoperative chemoradiation is contemplated, in most low and middle-income countries the cost of drugs is usually covered by patients who may found it prohibitive. A similar situation can be encountered regarding the availability of local or regional cancer centers equipped with surgical rooms and intensive care units required for radical surgical procedures. In both cases, qualified medical and surgical oncologists may also be insufficient or unavailable at all. Nevertheless, to have treatment options when the standard therapy is either not available of difficult to reproduce in particular settings is highly desirable with the potential to save lives that otherwise could be lost by the lack of adequate treatment.

Currently, a randomized prospective trial is ongoing in our Institution in which FIGO stage IB2/IIA receive EBRT at dose of 50 Gy concurrent with chemotherapy to then be randomized to either brachytherapy of surgery. The aim of the study is to demonstrate the superiority of the radical hysterectomy arms in terms of survival based on the fact that definitive chemoradiation (external beam and brachytherapy) is unable to sterilize pelvic lymph nodes in at least 15% of patients which can be removed by the surgery.

## Competing interests

The authors declare that they have no competing interests.

## Authors' contributions

LC, AGA, BBP and VF participated in data collection and analysis of results. MC, DC, LR, JC and AG participated in data analysis and substantial contribution to the manuscript. ADG conceived and wrote the manuscript.
